# Neural Stem Cell Transplant-Induced Effect on Neurogenesis and Cognition in Alzheimer Tg2576 Mice Is Inhibited by Concomitant Treatment with Amyloid-Lowering or Cholinergic **α**7 Nicotinic Receptor Drugs

**DOI:** 10.1155/2015/370432

**Published:** 2015-07-15

**Authors:** Anna M. Lilja, Linn Malmsten, Jennie Röjdner, Larysa Voytenko, Alexei Verkhratsky, Sven Ove Ögren, Agneta Nordberg, Amelia Marutle

**Affiliations:** ^1^Department of Neurobiology, Care Sciences and Society, Center for Alzheimer Research, Division of Translational Alzheimer Neurobiology, Karolinska Institutet, Karolinska University Hospital Huddinge, 141 57 Huddinge, Sweden; ^2^Faculty of Life Sciences, The University of Manchester, Manchester M13 9PT, UK; ^3^Department of Neuroscience, Karolinska Institutet, 171 77 Stockholm, Sweden; ^4^Department of Geriatric Medicine, Karolinska University Hospital Huddinge, 141 57 Huddinge, Sweden

## Abstract

Stimulating regeneration in the brain has the potential to rescue neuronal networks and counteract progressive pathological changes in Alzheimer's disease (AD). This study investigated whether drugs with different mechanisms of action could enhance neurogenesis and improve cognition in mice receiving human neural stem cell (hNSC) transplants. Six- to nine-month-old AD Tg2576 mice were treated for five weeks with the amyloid-modulatory and neurotrophic drug (+)-phenserine or with the partial *α*7 nicotinic receptor (nAChR) agonist JN403, combined with bilateral intrahippocampal hNSC transplantation. We observed improved spatial memory in hNSC-transplanted non-drug-treated Tg2576 mice but not in those receiving drugs, and this was accompanied by an increased number of Doublecortin- (DCX-) positive cells in the dentate gyrus, a surrogate marker for newly generated neurons. Treatment with (+)-phenserine did however improve graft survival in the hippocampus. An accumulation of *α*7 nAChR-expressing astrocytes was observed around the injection site, suggesting their involvement in repair and scarring processes. Interestingly, JN403 treatment decreased the number of *α*7 nAChR-expressing astrocytes, correlating with a reduction in the number of DCX-positive cells in the dentate gyrus. We conclude that transplanting hNSCs enhances endogenous neurogenesis and prevents further cognitive deterioration in Tg2576 mice, while simultaneous treatments with (+)-phenserine or JN403 result in countertherapeutic effects.

## 1. Introduction

In recent years expectations have been raised that stem cell replacement therapy could be used as a therapeutic approach to compensate for lost or damaged neuronal cells and to restore functionality in brain areas affected by chronic neurodegenerative diseases, such as Alzheimer disease (AD). The accumulation of amyloid-*β* (A*β*) and the formation of neurofibrillary tangles during AD pathogenesis are accompanied by cholinergic deficits involving a significant loss of nicotinic acetylcholine receptors (nAChRs), neuroinflammatory changes, and reduced growth factor production in the brain. These neurodegenerative processes alter the brain microenvironment resulting in neuronal dysfunction and ultimately in cognitive decline [1].

The hippocampus is affected early in the disease, and this region is one of only two known neurogenic niches of the adult brain [2] where new neurons are generated from neural stem/progenitor cells [3]. We have earlier proposed that the pathophysiological environment in the AD brain could have adverse effects on neurogenesis [3, 4], and it is suggested that the memory deficits observed in AD may be linked to alterations in hippocampal neurogenesis as reviewed in [5–7]. How neurogenesis is linked to cognitive function and whether stimulating regenerative mechanisms in the brain could restore or prevent further deterioration of cognition in the disease are actively being investigated. So far, observations from experimental studies in animal models of AD have provided supportive evidence that transplanted murine neural precursor or stem cells demonstrate neurotrophic, neuroprotective, and immunomodulatory potencies; these cells survive and migrate as normal, differentiate into neurons, astrocytes, and oligodendrocytes, and ameliorate learning and memory deficits in the recipient [8].

Moreover, we and others have earlier shown that pharmacological interventions can mobilize endogenous stem/progenitor cells in the hippocampus. Treatment with the drugs galantamine or memantine that are used to treat AD in clinical practice, as well as the candidate drugs (−)- and (+)-phenserine, increased neurogenesis* in vitro* in primary murine cortical cultures and* in vivo* in animal models of the disease [9–11].

In the current study, we applied a combinational therapeutical approach to investigate whether drugs with specific and distinct mechanisms of action on A*β* production and *α*7 nAChRs, respectively, could enhance neurogenesis and improve memory in human neural stem cell- (hNSC-) transplanted transgenic mice overexpressing the human amyloid precursor protein with the double Swedish mutation, Tg2576. This animal model is phenotypically similar to the prodromal stage of AD, and, at 6 to 9 months of age, Tg2576 mice demonstrate elevated levels of A*β* oligomers in the brain but no A*β* plaques, increased astrogliosis as well as impairment of synaptic function, and learning and memory deficits [12–15]. The study mice underwent intrahippocampal transplantation with 5 weeks' concurrent treatment with either (+)-phenserine, the partial *α*7 nAChR agonist JN403, or vehicle. We hypothesized that drug treatment could potentiate the effects of hNSC transplantation and the rationale for using these two drugs was based on previous reports of both amyloid-lowering and neurotrophic effects for (+)-phenserine [16, 17] and improvement in learning and memory following the stimulation of *α*7 nAChRs with selective cholinergic agonists including JN403 [18, 19]. Spatial learning and memory were tested both before and after intervention with transplantation and drug treatment. The subsequent effects on hNSC transplant survival, endogenous neurogenesis, and the involvement of a subpopulation of astroglia, the *α*7 nAChR-expressing astrocytes in the neurogenic niche of the dentate gyrus (DG), were determined.

## 2. Materials and Methods

### 2.1. Human Neural Stem Cell Culture

hNSCs were purchased from Lonza (Walkersville, MD, USA) and cultured in neural progenitor maintenance medium according to instructions from the manufacturer. These cells have previously been characterized* in vitro* [20]. For cellular differentiation* in vitro*, neurospheres were plated onto laminin-coated cover slips and cultured in DMEM F12 and neurobasal medium (1 : 1) supplemented with B27 (1 : 50) and 1% penicillin/streptomycin (Invitrogen, La Jolla, CA, USA). The cells were exposed weekly for a total of 28 days to extract from human AD autopsy frontal cortex (AD) containing 0.43 pM total A*β* or from a human healthy control (HC) diluted equivalently as the extract with 0.43 pM A*β* or to vehicle. In parallel experiments, cells were pretreated with 10^−7^ M JN403 or 10^−7^ M (+)-phenserine 24 hours prior to the addition of AD extract. For* in vitro* differentiation experiments, two independent experiments with four replicate samples for each treatment were used for analysis.

### 2.2. Tg2576 Mouse Cortical Primary Neurons

Primary neurons were isolated from the cerebral cortex of Tg2576 mouse embryos at embryonic day E17. The tissues were triturated to single cell suspension and plated onto poly-D-lysine-coated cover slips and cultured in neurobasal medium without glutamine and were supplemented with B27 (1 : 50), 100x Glutamax (1 : 400), and 1% penicillin/streptomycin (Invitrogen, La Jolla, CA, USA). The cells were exposed weekly to either 10^−7^ M JN403, 10^−7^ M (+)-phenserine, or vehicle following plating at the density 2 × 10^5^ cells/cm^2^. After 21 days in culture, the cells were washed with PBS and used for immunocytochemistry. Three biological replicates of each treatment were used for analysis.

### 2.3. Animals

Tg2576 mice expressing the APP Swedish mutation (APPSWE2576Kha), aged 5–7 months (3 females (F), 3 males (M)) and 6–9 months (17 F, 13 M), were obtained by backcrossing B6SJL (F1) females (Taconic) at the Karolinska Institutet animal care facility, as previously described [15]. Age-matched wild type littermates (3 F, 3 M) were used as control animals in the pilot study assessing memory in Tg2576 mice with the Morris water maze (MWM) navigation task. All mice were housed in enriched cages with a 12-hour light-dark cycle and access to food and water* ad libitum*. All experimental procedures complied with the guidelines and regulations of the Swedish National Board for Laboratory Animals and the Regional Ethics and Animal Research Committee at Karolinska Institutet approved the study protocol.

### 2.4. Drug Treatment

Tg2576 mice aged 6–9 months were divided into three treatment groups and given intraperitoneal injections of 0.3 mg/kg JN403 (*n* = 5) or 25 mg/kg (+)-phenserine (*n* = 7) solubilized in physiological saline solution or vehicle (physiological saline solution) (*n* = 18) once daily for 1 week. To monitor potential adverse drug reactions, JN403 was initially administered at dosages of 0.01 mg/kg (days 1-2) and 0.1 mg/kg (days 3-4) before reaching the full dose from day 5.

### 2.5. hNSC Transplantation

Cells were triturated, counted, and diluted with cell medium (vehicle). Tg2576 mice were anesthetized using a constant flow of 4% isoflurane and kept warm under a heating lamp throughout the transplantation procedure. The head of each mouse was fixed using ear and tooth bars before a skin incision into the skull bone was made using a 0.7 mm steel burr (Meisinger, Neuss, Germany) with the following coordinates relative to the bregma: AP −2.06, ML ±1.75, and DV −1.75 mm. Using a 26-gauge microsyringe (ILS Microsyringes, Stützerbach, Germany), approximately 25,000 cells per hemisphere were injected in a total volume of 1 *μ*L into the hippocampal DG of each Tg2576 mouse and the needle was thereafter kept in the injection site for approximately 30 seconds to allow the volume to diffuse. The wound was then sutured with metal clips. Lidocaine was used for local anesthesia during the procedure and 0.32 M sucrose solution was injected subcutaneously after surgery. The animals were monitored daily for body weight and proper healing of the incision site after surgery. No immunosuppressants were used, and no symptoms indicative of a reaction to the transplant were observed within the time frame investigated.

Drug treatment was interrupted on the day of transplantation and the mice were allowed a 3-day recovery period before drug treatment was continued for an additional 4 weeks. Demographic information of all the mice included in the transplantation is provided in Supplementary Table  1 (see Supplementary Material available online at http://dx.doi.org/10.1155/2015/370432) and the study outline is illustrated in Figure 2(a).

### 2.6. Morris Water Maze

First, a pilot study was conducted in which 5–7-month-old Tg2576 mice and their age-matched wild type littermates underwent testing for spatial learning and memory in the MWM task. The escape latency (time to reach the hidden platform) for Tg2576 mice and age-matched wild type littermates was compared during acquisition trials of 60 seconds' duration, which were performed 6 times a day at 10-minute intervals for 4 days. Both the latency to the center of the platform (time to reach the former platform location) and the number of platform crossings were measured.

Later, in the follow-up acquisition study, the Tg2576 mice (vehicle- or hNSC-transplanted and saline- or drug-treated) now aged 6–9 months underwent 60-second trials 4 times a day at 15-second intervals for 5 days. To assess retention of spatial memory 24 hours after the last acquisition trial, the platform was removed and the mice were tracked in the pool for 60 seconds in a probe trial. Both the latency to the center of the platform and the time spent in the target quadrant were measured. The mice were randomly placed in one of four fixed positions around the wall of a 1 m diameter round swimming pool. During the acquisition period, the mice learned the location of a hidden platform (9 × 9 cm), which was placed 1 cm under the water surface and 25 cm from the wall. The location of the platform was learned through visual cues (wall posters) on the walls around the pool. The water temperature was maintained at 21°C ± 2°C and the water was made opaque (white) to contrast the dark color of the mice from the black pool. The mice were habituated a day before the first acquisition day by letting them swim in the pool for 60 seconds without the platform. The platform location was fixed during the whole acquisition phase. Mice that did not find the platform within 60 seconds were aided to stay on the platform for 10 seconds to assist the learning process. The behavior of the mice in the MWM task was recorded by an automated video-tracking system (Ethovision). To evaluate the differences in memory retention between the groups in the probe trial, the baseline values for latency were subtracted from the follow-up latency values (Δ latency).

Two mice (one in the hNSC-transplanted and saline-treated group, as well as one in the hNSC-transplanted and (+)-phenserine treated group) could not be detected by the automated video-tracking system because of their color, and these mice were therefore omitted from the MWM tests.

### 2.7. Tissue Processing for Biochemical Analyses

Within 12–16 hours of completing the final behavioral assessments the animals were anesthetized with a 1 : 1 ketamine/xylazine mixture (100 mg/kg ketamine and 20 mg/kg xylazine) and sacrificed by transcardial perfusion with PBS buffer (pH 7.4). The brain from each animal was separated into hemispheres; the left hemisphere was snap frozen on dry-ice, and the right hemisphere was postfixed in buffered 4% paraformaldehyde and then transferred to a 0.32 M sucrose cryoprotectant solution for 24 h at 4°C. The brain hemispheres were stored at −80°C until further analysis. The left-brain hemispheres were subsequently used in the biochemical assays, and the right hemispheres were cryostat sectioned and used in immunohistochemistry studies.

### 2.8. Immunohistochemistry

For staining of differentiated hNSCs in culture, cells were incubated with rabbit anti-human glial fibrillary acidic protein (GFAP, Dako, Glostrup, Denmark), mouse anti-*β*III-tubulin (Sigma, St. Louis, MO, USA), or mouse polyclonal anti-microtubule-associated protein 2 (MAP2, Sigma, St. Louis, MO, USA). All primary antibodies were diluted 1 : 250. Thereafter, cells were incubated with secondary Alexa Fluor conjugated antibodies (1 : 500, donkey anti-mouse Alexa Fluor 546, or donkey anti-rabbit Alexa Fluor 488; Molecular Probes, Eugene, OR, USA). Cells immunopositive for DAPI and GFAP or *β*III-tubulin or the number of dendritic branches per DAPI and MAP2 immunopositive cell was counted in three random fields per cover slip (six fields per treatment under a Nikon E800 microscope).

In order to obtain comparable results between the different animals, for each individual animal three coronal sections from the same hippocampal regions were selected for immunostaining. Newly generated neurons in the hippocampus were labeled with an antibody for the microtubule-associated protein Doublecortin (DCX) (goat anti-DCX 1 : 200, Santa Cruz, Heidelberg, Germany) according to the method described previously by Lilja et al. [10]. DCX immunoreactivity was analyzed in three serial sections for each animal. All DCX+ cells in the DG were quantified blindly, in which the total number of DCX+ cells, the proportion of DCX+ cells with dendrites, and the number of branch points on the DCX+ cells with more pronounced dendritic branching were counted.

To investigate graft survival and the fate of transplanted hNSCs in the DG, fluorescent immunohistochemical analysis was carried out on serial sections from each animal using primary antibodies polyclonal rabbit antiglial fibrillary acidic protein (GFAP) (1 : 500, Dako, Glostrup, Denmark), polyclonal rabbit anti-microtubule-associated protein 2 (MAP2) (1 : 500, Millipore, Temecula, CA, USA), and monoclonal mouse anti-human nuclei (hNuclei) (1 : 200, Millipore, Temecula, CA, USA). Secondary antibodies were conjugated with Alexa Fluor (AF; AF 488 donkey anti-rabbit and AF 546 donkey anti-mouse, 1 : 500, Molecular Probes, Eugene, OR, USA). In control experiments, the primary antibody was omitted and a blocking buffer was substituted to verify the specificity of the secondary antibodies. The specificity of hNuclei antibody staining was also assessed in mice that had not received any hNSCs implants. Immunoreactive cells within the DG (including the subgranular zone and granular and polymorph layers) were counted in three serial sections from each animal.

The number of GFAP/*α*7 nAChR immunopositive cells was evaluated on three serial coronal sections per animal after consecutive incubations with primary antibodies (1 : 500 rabbit anti-GFAP, Dako; 1 : 100 rat anti-*α*7 nAChR, Abcam) and secondary antibodies (1 : 200 goat anti-rabbit IgG-Fab horseradish peroxidase- (HRP-) conjugated; goat anti-rat IgG-Fab HRP-conjugated, Invitrogen, La Jolla, CA, USA). Immunoreactivity was visualized using peroxidase substrate kits (DAB and SG, Vector, resp.). The results were quantified at 20x magnification in a prespecified area (325 *μ*m × 435 *μ*m = 141375 *μ*m^2^) using software ImageJ (NIH, Bethesda, MD, USA).

Nonfluorescent staining was evaluated under a light microscope (Leica, Wetzlar, Germany) with an attached ProgRes SpeedXT core5 video camera and an image analysis system (ProgRes Capture Pro2.8.8). Fluorescent staining was evaluated under a Nikon E800 microscope at 20x magnification using Adobe Photoshop CS5 software (San José, CA, USA).

### 2.9. A*β*_40_ and A*β*_42_ ELISA

Frontal cortices and hippocampi were extracted from the left hemisphere, immersed in PBS buffer (pH 7.4), and homogenized. Samples were diluted 1 : 25 in BSAT-DPBS (Dulbecco's PBS with 5% BSA, 0.03% Tween-20, and 1x protease inhibitor cocktail). A*β*_40_ and A*β*_42_ levels were measured using a sandwich ELISA assay (Invitrogen, Camarillo, CA, USA), according to the manufacturers' instructions, and the absorbance was read at 450 nm (Tecan Infinite M1000).

### 2.10. Statistical Analysis

GraphPad Prism 5.0 or 6 (GraphPad Software, Inc.) was used for all statistical analyses. Data were analyzed using the Mann-Whitney test for comparison between two groups and the Kruskal-Wallis one-way ANOVA test by rank followed by Dunn's* post hoc* test for comparison of >2 groups for both* in vitro* and* in vivo* analyses. Repeated measures ANOVA was applied for comparison of wild type and Tg2576 mice in the acquisition tests in Morris water maze. Spearman's rank correlation was used in the correlation analysis, which was visualized graphically using simple regression analysis. The data are presented as means ± standard error of the mean (SEM). *p* values < 0.05 were considered to be significant.

## 3. Results

### 3.1. JN403 Exerts Neuroprotective Effects on hNSC-Derived Neurons in Culture

Neurotrophic actions of the *α*7 nAChR partial agonist JN403 and the A*β* modulatory drug (+)-phenserine were assessed by examining the survival, differentiation, and maturation of progenitor cells* in vitro*. JN403 protected hNSC-derived *β*III-tubulin marked neurons against brain extract from a patient with AD containing various A*β* species (Figure S1A). On the other hand, JN403 or (+)-phenserine did not significantly alter the number and/or pattern of MAP2-positive Tg2576 cortical neurons in culture (Figure S1B).

### 3.2. Tg2576 Mice Demonstrate Impaired Spatial Memory Navigation

**Figure 1:**
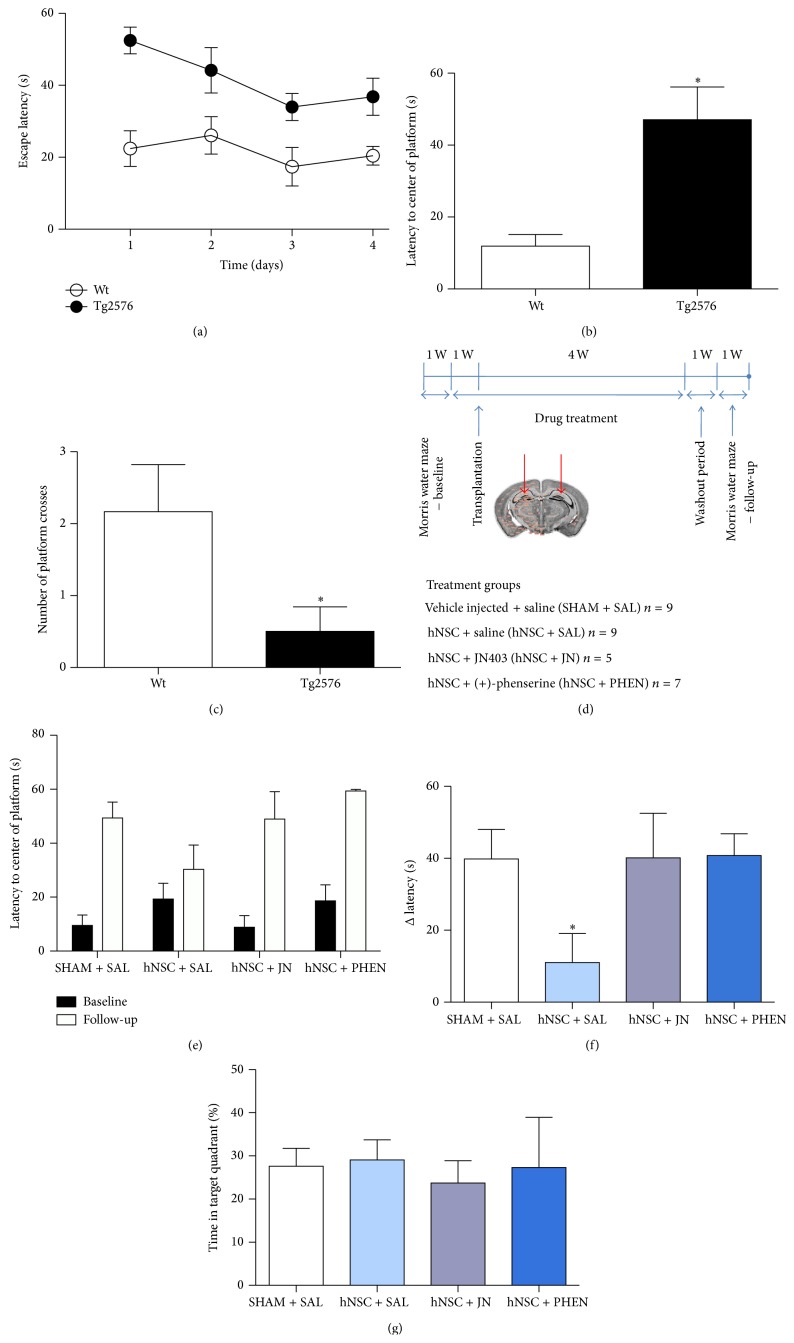
Intrahippocampal hNSCs transplantation affects spatial navigation memory in Tg2576 mice. Learning and memory were assessed in 5- to 7-month-old wild type (wt, *n* = 6) and Tg2576 (APPswe, *n* = 6) mice in the pilot Morris water maze task. (a) Escape latency during 4 days of acquisition training. (b) Latency to the former location of the platform or (c) the number of platform crosses in a probe test 24 hours later. ^*∗*^ *p* < 0.05 compared to wild type. The data are expressed as means ± SEM. (d) The experimental design of the study with 6- to 9-month-old Tg2576 mice (*n* = 30) comprised (i) Morris water maze tests with 5 days of acquisition followed 24 hours later by a probe memory test, (ii) daily intraperitoneal injections of 0.3 mg/kg JN403 or 25 mg/kg (+)-phenserine for 5 weeks in total, (iii) bilateral hippocampal injections of ~25,000 hNSCs/hemisphere after 1 week of drug treatment, (iv) repetition of the Morris water maze tasks 5 weeks after transplantation, and (v) sacrifice by transcardial perfusion with PBS. (e) Time taken to find the former platform location in the baseline and follow-up test. (f) Differences in time taken to find the former platform location (Δ latency; follow-up probe test values minus baseline probe values). (g) Percent time spent in the target quadrant where the platform was located. ^*∗*^ *p* < 0.05 compared to SHAM + SAL. The data are expressed as means ± SEM.

Hippocampal-dependent spatial learning and memory in Tg2576 mice (*n* = 6) and age-matched nontransgenic littermates (wild type, *n* = 6) were tested in the MWM navigation task. During the four days of acquisition, training of Tg2576 mice resulted in an expected learning curve, whereas training of the wild type mice resulted in a very shallow learning curve where escape latencies were similar over time (Figure 1(a)). The latency to the former platform location in the probe trial 24 hours after the last day of training (day 4) was also significantly longer for Tg2576 mice than for wild type mice (47.1 s compared to 11.9 s, *p* < 0.05, Mann-Whitney test; Figure 1(b)). Wild type mice crossed the former location of the platform more often than the Tg2576 mice (*p* < 0.05, Mann-Whitney test; Figure 1(c)). In contrast, the swimming velocity and the total distance the mice swam during the acquisition period (Figures S2A and S2B) did not differ significantly between the groups. Differences in escape latency between female and male mice were also assessed and no significant differences appeared.

### 3.3. Intrahippocampal hNSC Transplantation Prevents Memory Deterioration in Tg2576 Mice

To ensure that there were no differences in learning and memory among the Tg2576 mice that could be attributed to the age range (6–9 months), the mice were subjected to a baseline MWM test before the onset of treatment (an overview of the study design is provided in Figure 1(d)). No significant differences in test results were observed among the different treatment groups (at baseline or at follow-up) during the 5 days of acquisition training. In general, improvements in escape latencies were minor and resulted in similar shallow learning curves as those observed during training of wild type mice in the pilot study. In the 24-hour probe trial, there were no differences in latency baseline values between groups (Figure 1(e); *p* = 0.26, Dunn's test). The cohort of hNSC-transplanted Tg2576 mice had a memory performance comparable to their baseline values at the MWM follow-up test, whereas the other treatments groups deteriorated compared to baseline (Figure 1(e)). The progression of pathology as well as extensive neurosurgery could have influenced the performance at follow-up. The hNSC-transplanted mice found the former platform location significantly faster than SHAM-transplanted mice in the MWM follow-up test (Δ latency 11.0 s and 39.9 s, resp.; *p* < 0.05, Dunn's test; Figure 1(f)). The hNSC-transplanted Tg2576 mice treated with either JN403 or (+)-phenserine showed no memory improvements compared to SHAM-transplanted mice (Figure 1(f)).

The standard measurement of memory is the percent time spent in the target quadrant compared with the other quadrants, rather than the latency to reach the former location of the platform. The different groups of mice spent an equally short time in the target quadrant, thus showing a low persistence, which is not unusual for mice (Figure 1(g)). Swimming velocity or the total distance swum did not differ among Tg2576 mice in the various groups (Figures S2C and S2D).

### 3.4. hNSC Transplantation Increases Endogenous Neurogenesis in the Dentate Gyrus

**Figure 2:**
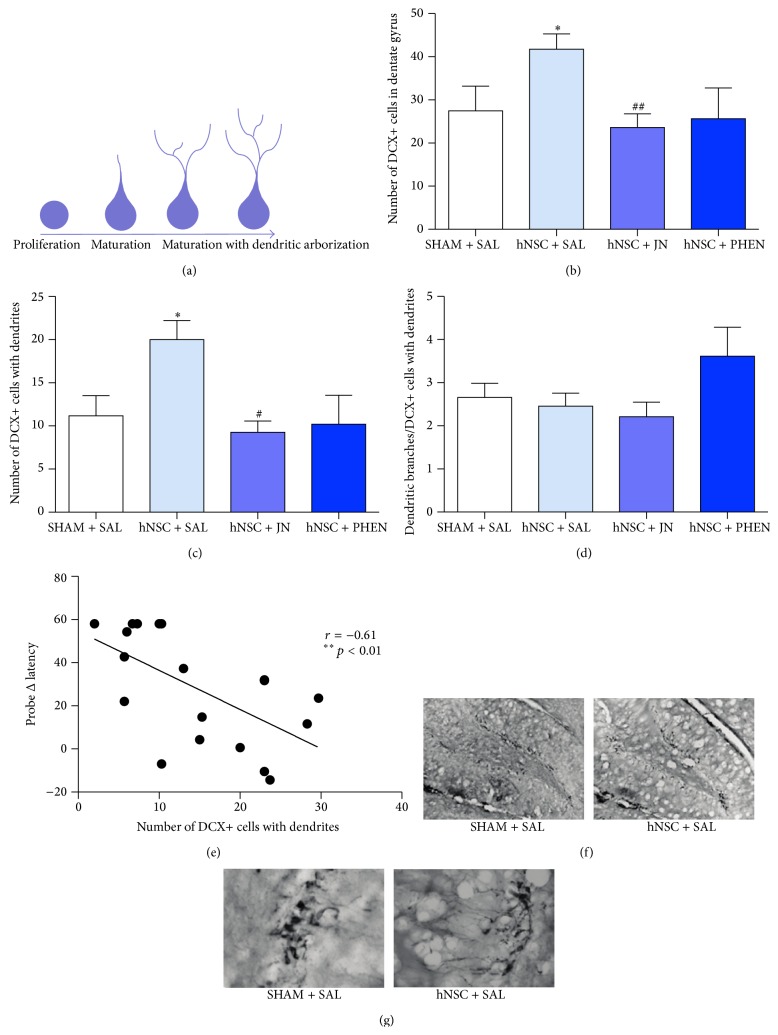
hNSC transplantation increases the number and the maturation of new neurons in the dentate gyrus. (a) Doublecortin- (DCX-) labeled cells in the dentate gyrus (DG) of Tg2576 mice were characterized according to their numbers and the level of maturation and dendritic arborization. (b) The total number of DCX+ cells, (c) the number of DCX+ cells possessing dendrites, and (d) the average number of dendritic branches on DCX+ cells in the DG in mice receiving SHAM (vehicle only) transplantation and saline (SHAM + SAL) or mice receiving hNSC transplantation and saline (hNSC + SAL), JN403 (hNSC + JN), or (+)-phenserine (hNSC + PHEN). (e) Correlation between the number of DCX+ cells with dendrites and the difference in time taken to find the position of the platform (Δ latency; follow-up probe test values minus baseline acquisition values) in the Morris water maze task in groups of Tg2576 mice treated with hNSC + SAL, hNSC + JN, and hNSC + PHEN (*r* = −0.61; ^*∗∗*^ *p* < 0.01, *n* = 18). Representative images of immunostaining with DCX+ cells in the DG of TG2576 mice at (f) 10x and (g) 20x magnification. ^*∗*^ *p* < 0.05 compared to SHAM + SAL, ^#^ *p* < 0.05 and ^##^ *p* < 0.01 compared to hNSC + SAL. The data are expressed as means ± SEM.

The early neuronal marker DCX is transiently expressed during a period that extends from a proliferative progenitor cell stage to a postmitotic phase at which DCX-positive cells differentiate into mature neurons with increasing dendritic arborization in the DG, as illustrated in Figure 2(a). We thus characterized neural stem/progenitor cells DCX+ cells as an index of neurogenesis. These cells may derive from the resident pool of progenitor cells in the hippocampal DG or from the exogenous source of transplanted hNSCs. DCX+ cells were detected exclusively in the subgranular layer, while cells positive for the marker human nuclei (hNuclei+ cells), as described in the following section, were observed mainly in the polymorph layer of the DG. This indicates that the DCX+ cells detected were mainly derived from the endogenous stem cell pool. Immunohistochemical analysis demonstrated that hNSC transplantation in Tg2576 mice augmented endogenous neurogenesis, as increases in both the total number of DCX+ cells (56% higher) and the number of maturing DCX+ cells (82% higher) were observed in the DG, compared to SHAM transplantation (both *p* < 0.05, Dunn's test; Figures 2(b) and 2(c)).

Treatment with JN403 and (+)-phenserine, however, counteracted the beneficial effects of hNSC transplantation on endogenous neurogenesis. Moreover, JN403 reduced the total number of DCX+ cells (43% lower, *p* < 0.01, Dunn's test) as well as the number of DCX+ cells with dendrites (55% lower; *p* < 0.05, Dunn's test) compared to vehicle-treated hNSC-transplanted mice. Treatment of hNSC-transplanted mice with (+)-phenserine did not significantly alter the number of DCX+ cells (Figures 2(b) and 2(c)). There were no significant differences in the number of dendritic branches per DCX+ cell among the different groups (Figure 2(d)).

**Figure 3:**
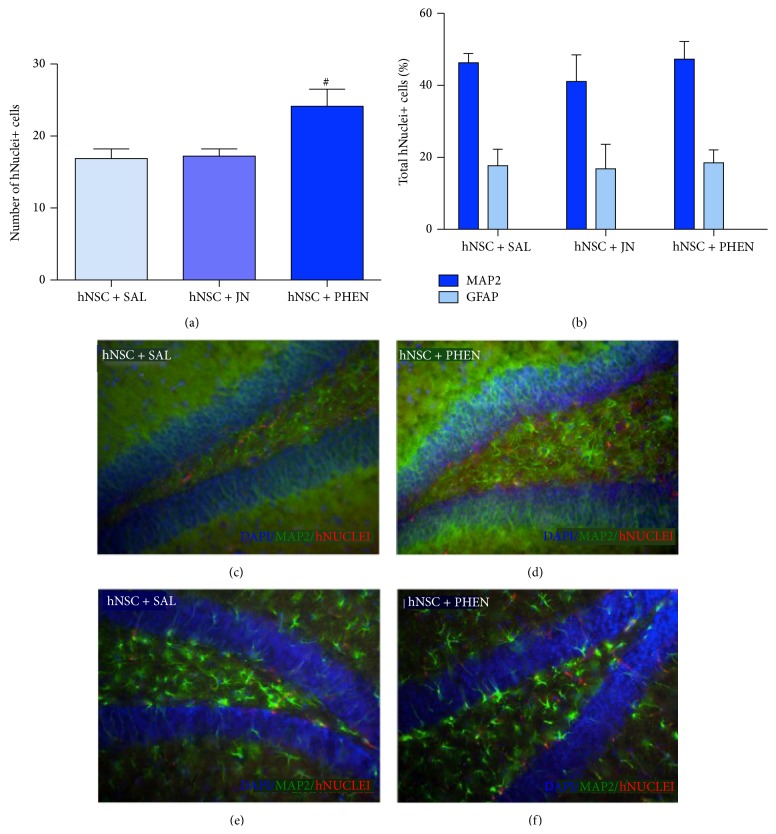
Treatment with (+)-phenserine increases the survival of transplanted hNSCs in the dentate gyrus of Tg2576 mice. (a) The total number of cells labeled with anti-human nuclei (hNuclei) and with 4′,6-diamidino-2-phenylindole (DAPI) nuclear counterstain following transplantation of hNSCs into the dentate gyrus (DG) in Tg2576 mice. (b) The proportion of cells showing immunoreactivity for microtubule-associated protein 2 (MAP2) or glial fibrillary acidic protein (GFAP), normalized to the total number of hNuclei+ cells (%) in the DG of Tg2576 mice. (c-d) Representative images of cells stained with hNuclei (red) and with MAP2 (green) in the DG of hNSC-transplanted mice treated with (c) saline (hNSC + SAL) or (d) (+)-phenserine (hNSC + PHEN) at 20x magnification. (e-f) Representative images of cells stained with hNuclei (red) and with GFAP (green) in the DG of hNSC-transplanted mice treated with (e) saline or (f) (+)-phenserine at 20x magnification. Cell nuclei were visualized with DAPI. ^#^ *p* < 0.05 compared to hNSC + SAL. The data are expressed as means ± SEM.

The number of DCX+ cells with dendrites inversely correlated with measures of the Δ latency obtained in the probe trial (*p* < 0.01, *r* = −0.61, Spearman's rank; Figure 3(e)), indicating that the increased maturation of newborn neurons in the DG following hNSC transplantation was related to an improvement in hippocampus-dependent memory. Representative images of DCX+ cells in the DG of hNSC- and SHAM-transplanted Tg2576 mice are shown in Figures 2(f) and 2(g).

### 3.5. (+)-Phenserine Treatment Increases the Survival of hNSC-Derived Neurons in the Hippocampus of Tg2576 Mice

Chronic treatment with (+)-phenserine increased the survival of engrafted hNSCs in the DG of Tg2576 mice as demonstrated by human nuclei antibody labeling. The number of hNuclei+ cells was larger in Tg2576 mice treated with (+)-phenserine than in those receiving saline (43% increase; *p* < 0.05, Dunn's test), while there were no differences in numbers of hNuclei+ cells between JN403- and saline-treated mice (Figure 3(a)). Costaining hNuclei+ cells with MAP2 or GFAP, markers for mature neurons and astrocytes, respectively, revealed that approximately 70% of the engrafted hNSCs were hNuclei+/MAP2+ and 20% were hNuclei+/GFAP+. A similar differentiation profile was observed in all Tg2576 cohorts, irrespective of the treatment paradigm (Figures 3(b) to 3(f)).

### 3.6. (+)-Phenserine Treatment Reduces A*β*_40_ Levels in the Brains of hNSC-Transplanted Tg2576 Mice

We have previously reported a reduction in A*β*_42_ levels in the frontal cortex in 4–6-month-old Tg2576 mice treated with (+)-phenserine [11]. In this study, a reduction in A*β*_40_ levels was measured in the frontal cortices and hippocampi of hNSC-transplanted Tg2576 mice treated with (+)-phenserine, but not in the cohort of mice treated with JN403 or saline. In comparison to SHAM-transplanted, saline-treated mice, a significant reduction in A*β*_40_ levels, but not in A*β*_42_ levels, was measured in the frontal cortex (29% reduction; *p* < 0.05, Dunn's test; Figure S3A) and hippocampus (42% reduction; *p* < 0.05, Dunn's test; Figure S3B) of hNSC-transplanted mice treated with (+)-phenserine. No significant changes in A*β*_40_ and in A*β*_42_ levels were observed in saline- or JN403-treated hNSC-transplanted Tg2576 mice versus SHAM-transplanted, saline-treated mice.

### 3.7. Association between Endogenous Neurogenesis and the Presence of *α*7 nAChR-Expressing Astrocytes in the Dentate Gyrus of hNSC-Transplanted Tg2576 Mice

**Figure 4:**
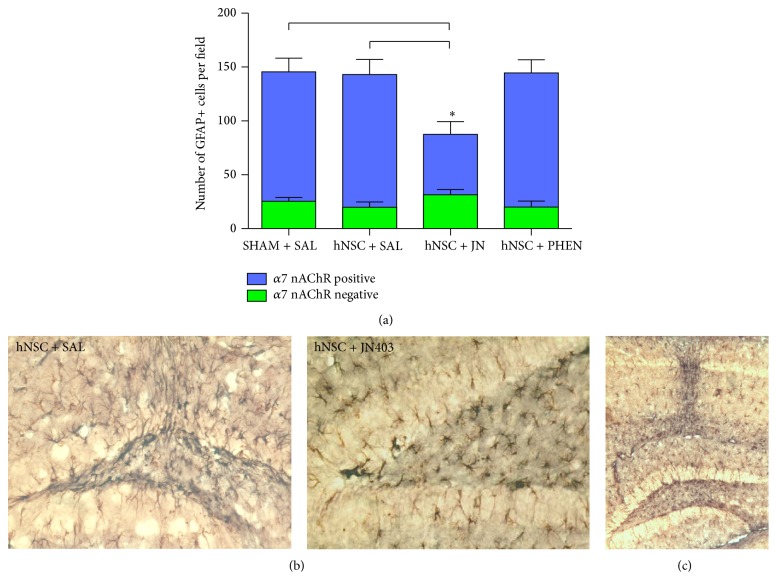
Treatment with the cholinergic *α*7 nicotinic receptor agonist JN403 decreases the number of *α*7 nicotinic receptor-expressing astrocytes in the dentate gyrus. (a) The number of *α*7 nicotinic receptor/glial fibrillary acidic protein-positive (nAChR+/GFAP+) cells and the number of *α*7 nAChR-negative/GFAP+ cells in the dentate gyrus of Tg2576 mice that received SHAM (vehicle) transplants and saline (SHAM + SAL) or hNSC transplants and saline (hNSC + SAL), JN403 (hNSC + JN), or (+)-phenserine (hNSC + PHEN). (b) Representative images of *α*7 nAChR+/GFAP+ cells (black) and *α*7 nAChR-negative/GFAP+ cells (brown) in hNSC + SAL and hNSC + JN mice. (c) Representative image of *α*7 nAChR+/GFAP+ cells (black) in the needle track after hippocampal injection. ^*∗*^ *p* < 0.05 compared to SHAM + SAL. The data are expressed as means ± SEM.

**Figure 5:**
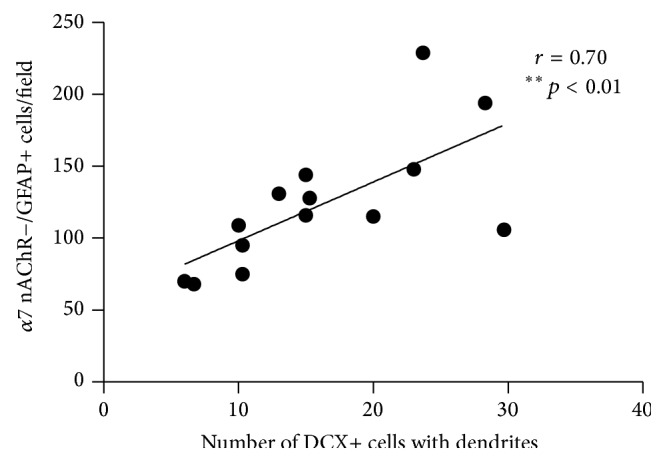
The number of newly generated neurons correlates with the proportion of *α*7 nicotinic receptor-expressing astrocytes in the dentate gyrus of hNSC-transplanted Tg2576 mice. The number of maturing Doublecortin- (DCX+-) labeled cells showed a significant positive correlation with the number of *α*7 nicotinic receptor/glial fibrillary acidic protein-positive (nAChR+/GFAP+) cells in the dentate gyrus of Tg2576 mice (*r* = 0.70, ^*∗∗*^ *p* < 0.01, *n* = 13). The data are expressed as the mean value for each parameter in the hNSC-transplanted, saline-treated mice (hNSC + SAL) and the hNSC-transplanted, JN403-treated mice (hNSC + JN).

Reactive astrogliosis and numerous *α*7 nAChR-expressing GFAP+ astrocytes were detected in the hippocampi of hNSC-transplanted Tg2576 mice. Significant reductions were observed in the total numbers of GFAP+ astrocytes (30% reduction, *p* < 0.05) and even greater decrease was seen in the number of astrocytes expressing *α*7 nAChRs (39-40% reduction; *p* < 0.05, Dunn's test) in the DG of hNSC-transplanted Tg2576 mice treated with JN403, compared with mice receiving SHAM or hNSC transplants and saline (Figures 4(a) and 4(b)). Treatment with (+)-phenserine did not alter the number of *α*7 nAChR-expressing astrocytes in the hNSC-transplanted DG compared to saline treatment in SHAM- or hNSC-transplanted mice (Figure 4(a)). A large number of *α*7 nAChR-expressing astrocytes were observed in the area of the DG surrounding the injection site (Figure 4(c)), suggesting the involvement of *α*7 nAChR-expressing astrocytes in repair and regenerative processes in the brain. In support of this, a significant positive correlation was found between *α*7 nAChR-expressing astrocytes and number of DCX+ cells with dendrites (*r* = 0.70; *p* < 0.01, Dunn's test; Figure 5) in hNSC-transplanted Tg2576 mice.

## 4. Discussion

### 4.1. hNSC Transplantation Enhances Endogenous Neurogenesis and Prevents Memory Deterioration in Tg2576 Mice

Transplantation of hNSCs increased endogenous neurogenesis in the DG, which correlated with improved hippocampal-dependent memory performance. The hNSC-transplanted mice had a memory performance comparable to baseline values, which might indicate stabilization of the disease, whereas the other treatment groups continued to deteriorate. It could be argued that extensive training (baseline and follow-up) may improve the performance. However, the memory deterioration could be a consequence of the progression of the disease. Although informative, the behavioral paradigm employed in this study has its limitations. Reversal learning in the MWM (the ability of the mice to find a new location of the hidden platform) has recently gained preference for the assessment of hippocampal-dependent memory in relation to neurogenesis [21]. However, subjecting the study mice to further assessments after they had undergone baseline, follow-up, and probe trial sessions would have been too exhausting and most likely had an impact on their performance. Another limitation in the behavioral testing is that we did not study visual discrimination learning. A solution to this would have been to include an initial visual test to assess eyesight. Despite these limitations, the findings presented here are in line with those of a previous study that showed that intrahippocampal transplantation of murine NSCs resulted in an increased secretion of brain-derived neurotrophic factor (BDNF) and synaptic density, as well as improved cognition in aged 3xTg-AD mice harboring a heavy A*β* plaque burden and neurofibrillary tangle pathology [22]. Several studies have reported an imbalance between the levels of precursor and mature BDNF (the former possessing proapoptotic features) as well as impaired BDNF signaling [23–27]. Furthermore, a decline in BDNF levels in the brain occurs with disease progression and is correlated with cognitive decline [25, 28]. Despite the promising potential for using BDNF as a biomarker to assess therapeutic effects, quantitative measurements of BDNF levels with ELISA-based techniques are difficult to perform as the current available antibodies lack sufficient sensitivity to distinguish mature forms of BDNF from pro-BDNF [29, 30]. We hypothesize that impaired neurotrophic support in the AD brain may be one of the underlying mechanisms through which endogenous neurogenesis is compromised preventing maturation of newborn neurons. Our current findings indicate that engraftment of hNSCs in Tg2576 mouse brains provides neurotrophic support to existing neurons in the neural circuitry as well as to endogenous stem cells residing in the DG.

### 4.2. (+)-Phenserine Treatment Decreases A*β* Levels and Enhances Graft Survival but Mitigates the hNSC-Mediated Beneficial Effects on Endogenous Neurogenesis and Cognition in Tg2576 Mice

(+)-phenserine treatment combined with hNSC transplantation in Tg2576 mice lowered both cortical and hippocampal A*β*_40_ levels and increased the survival of transplanted hNSCs in the hippocampi. However, this treatment combination failed to improve memory in Tg2576 mice. Surprisingly, we found that the administration of (+)-phenserine prevented the hNSC-induced increase in endogenous neurogenesis in Tg2576 mice. We have earlier demonstrated that (+)-phenserine augments neuronal differentiation of transplanted hNSCs in the brains of AD APP23 transgenic mice [4]. We have also reported that (+)-phenserine, in addition to its A*β* lowering action, exerts prosurvival effects on progenitor cells [10] and increases the dendritic arborization of hippocampal neurons [11]. Hence, the body of evidence that (+)-phenserine possesses neurotrophic characteristics rules out the possibility that (+)-phenserine per se inhibited neurogenesis but rather antagonized the trophic effects of hNSC transplantation.

The mitogen-activated protein kinases (MAPK) and protein kinase C signaling pathways have been identified as potential mediators of the neurotrophic actions of (+)-phenserine. These pathways are involved in a diverse repertoire of biological events including proliferation, differentiation, metabolism, motility, survival, and apoptosis [10]. Both MAPK and the downstream transcription factor CREB are important regulators of BDNF [31, 32] and share common signaling pathways with other key regulators of adult neurogenesis and NSCs, such as fibroblast growth factor-2, insulin-like growth factor-1, and vascular endothelial growth factor [33]. Hence, a possible explanation for the unexpected and somewhat ambiguous observations with (+)-phenserine in the present study could be that the simultaneous combination of transplantation and chronic (+)-phenserine administration interferes with hNSC signaling cascades, which could potentially lead to an inhibition of the neurotrophic effects of these cells on the brain environment that normally supports neurogenesis in Tg2576 mice. Identifying the underlying mechanisms warrants future investigation, yet our current findings raise the question of whether the dosage regimen or interval of (+)-phenserine administration in combination with hNSCs needs to be carefully considered in lieu of the different neurodegenerative stages in AD. Although hNSC transplantation induces beneficial effects, impaired graft survival as a result of the accumulation of pathological proteins in the AD brain should be considered, as has previously been demonstrated in grafted neurons in Parkinson patients [34]. Hence, to sustain the efficacy of this intervention, we propose that combination treatment with drugs that target the different pathological processes without interfering with the stem cell-mediated neurogenic effects should be administered at specific disease stages.

### 4.3. The *α*7 nAChR Agonist JN403 Impairs Neurogenesis and Cognition, Which Correlates with Downregulation of *α*7 nAChR-Expressing Astrocytes

Neurotrophic effects for (+)-phenserine have been observed in multiple studies [10, 16, 17], whereas similar effects of JN403 remained to be explored. To this end, we performed initial* in vitro* experiments to investigate the effects of JN403 on neuroprotection and on neuronal differentiation. JN403 increased the survival of hNSC-derived neurons exposed to brain extract containing various A*β* species, in line with the characteristic neuroprotective effects induced by treatment with *α*7 nAChR agonists [35–38].

Reactive gliosis is a complex and multistage process and plays an important role in the progression and resolution of brain pathology. Astrocytes are a diverse group of cells with many subclasses that carry out a wide range of functions in the CNS including the homeostatic response, limiting damage to the brain through formation of a glial scar, and removal of neurotoxic pathogens and assist in postlesion regeneration of the neural networks [39]. It has become a matter of growing interest and ongoing investigation whether all astrocytes or only certain types of astrocytes in the brain are involved in the regulation of cognitive processes. Recent* in vivo* observations with positron emission tomography (PET) imaging suggest that astroglial reactivity correlates with the preservation of cognitive function in patients with mild cognitive impairment and prodromal AD [40]. The *α*7 nAChR-expressing astrocytes are reported to be associated with the pathophysiological processes in AD, as increased numbers have been found surrounding *β*-amyloid plaques in postmortem brains of sporadic AD and APP Swedish mutation carriers [39]. In the current study, we detected a large number of *α*7 nAChR-expressing astrocytes surrounding the injection site in the DG of Tg2576 mice, indicative of a tissue response to the cell transplantation. In addition, a significant positive correlation was found between the numbers of *α*7 nAChR-expressing astrocytes and the numbers of newly generated neurons in the DG. These data suggest a role for this subclass of astrocytes in repair and scarring processes in the brain.

Inhibitory effects of chronic JN403 treatment on hNSC-induced neurogenesis and cognition were observed in Tg2576 mice. JN403 induced a downregulation in both the total number of astrocytes and the number of *α*7 nAChR-expressing astrocytes in the DG. It is possible that the duration of treatment with JN403 led to a suppression of the normal physiological and neuroprotective functions of *α*7 nAChR-expressing astrocytes.

Functional *α*7 nAChRs on astrocytes in hippocampal slices from rat brain have previously been demonstrated [41]. Considering the high Ca^2+^-permeability of *α*7 nAChRs, activation of these receptors would thus lead to increases in intracellular calcium levels in hippocampal *α*7 nAChR-expressing astrocytes to an extent that may significantly impact synaptic transmission and plasticity mechanisms. However, the *α*7 nAChRs also undergo rapid desensitization, and the pharmacodynamic effects and impact of nAChR agonist stimulation versus desensitization on cognition are still poorly understood. The *α*7 nAChRs on neurons and astrocytes may have different roles and may be differentially affected during the various stages of disease. Upregulation of neuronal *α*7 nAChR expression with A*β* accumulation is reported in the early stages of AD [42, 43] as well as in Tg2576 mice [44] and most likely reflects a compensatory response to maintain memory function. As the disease progresses, neuronal *α*7 nAChRs may either decrease [45] or increase, where hyperexcitable neuronal *α*7 nAChRs further exacerbate neurotoxicity and neurodegeneration [46]. Reconciling the findings from these studies would therefore suggest that the modulation of *α*7 nAChRs present on either neuronal or nonneuronal cells could induce either protective or toxic effects depending on the mode of agonist exposure and on the functional status of these receptors during the disease course. The expression of *α*7 nAChRs on neurons was not quantified in our study and we cannot rule out the possibility that JN403 may have exerted an antagonistic function on hippocampal-dependent memory functions, through mechanisms that also involved neuronal *α*7 nAChRs. The *α*7 nAChRs are present early during development and these receptors have also been detected on human stem cells [47–49], where they likely mediate effects of cholinergic signaling on stem cell survival/apoptosis, proliferation, differentiation, and maturation. An alternative mechanism is that JN403 interacts with the *α*7 nAChRs expressed on transplanted hNSCs with downstream consequences on *α*7 nAChR signaling pathways. These changes could in turn result in a modulation of the proposed trophic actions of the grafted cells and consequently affect endogenous neurogenesis and cognition.

The potential anti-inflammatory mechanisms and regulation of inflammatory processes in the brain caused by the stimulation *α*7 nAChRs with selective agonists should also be considered. Although these mechanisms are complex and not clearly understood, it is conceivable that JN403 stimulation of the *α*7 nAChRs on astrocytes modulates the anti-inflammatory response, where it is important that a certain innate basal level of *α*7 nAChR-expressing GFAP+ astrocytes is maintained in the hippocampus. In support of the latter, a recent study using a rodent model of Parkinson's disease showed that targeting *α*7 nAChRs induces anti-inflammatory effects via inhibition of astrocyte activation [38].

In conclusion, the present study reveals that hNSC transplantation stimulates regenerative processes in the brain. Furthermore, the findings indicate that hNSC transplantation can attenuate memory deterioration. In contrast, coadministration with either drug (+)-phenserine or JN403 inhibits the beneficial effects of hNSC infusion. The enhancement of endogenous neurogenesis in mice following transplantation shows a positive correlation with *α*7 nAChR-expressing astrocytes in the DG. Therapies that stimulate endogenous neurogenesis in AD brain could thus contribute to improvement of cognitive function.

## Supplementary Material

JN403 exerts neuroprotective effects on hNSC-derived neurons in culture Neurotrophic actions of the α7 nAChR partial agonist JN403 and the Aβ modulatory drug (+)-phenserine were assessed by examining the survival, differentiation and maturation of progenitor cells in vitro. JN403 protected hNSC-derived βIII-tubulin marked neurons against brain extract from a patient with AD containing various Aβ species (Fig. S1A). On the other hand, JN403 or (+)-phenserine did not significantly alter the number and/or pattern of MAP2-positive Tg2576 cortical neurons in culture (Fig. S1B).
The swimming velocity and the total distance the mice swam during the acquisition period did not differ significantly between the groups Hippocampal-dependent spatial learning and memory in Tg2576 mice (n=6) and age-matched non-transgenic littermates (wild type, n=6) were tested in the MWM navigation task. The swimming velocity and the total distance the mice swam during the acquisition period did not differ significantly between transgenic and wildtype animals (Figures S2A and S2B). Tg2576 mice (vehicle- or hNSC-transplanted and saline- or drug-treated), aged 6–9 months, underwent 60-second trials 4 times a day at 15-second intervals for 5 days. Swimming velocity or the total distance swum did not differ among Tg2576 mice in the various groups (Figure S2C and S2D).
(+)-Phenserine treatment reduces Aβ_40_ levels in the brains of hNSC-transplanted Tg2576 mice In this study, a reduction in Aβ40 levels was measured in the frontal cortices and hippocampi of hNSC-transplanted Tg2576 mice treated with (+)-phenserine, but not in the cohort of mice treated with JN403 or saline. In comparison to sham-transplanted, saline-treated mice, a significant reduction in Aβ40 levels, but not in Aβ42 levels, was measured in the frontal cortex (29 % reduction, p<0.05, Dunn's test, Figure S3A) and hippocampus (42 % reduction, p<0.05, Dunn's test, Figure S3B) of hNSC-transplanted mice treated with (+)-phenserine. No significant changes in Aβ_40_ and in Aβ_42_ levels were observed in saline- or JN403-treated hNSC-transplanted Tg2576 mice versus sham-transplanted, saline-treated mice.

## References

[1] Paterson D., Nordberg A. (2000). Neuronal nicotinic receptors in the human brain. *Progress in Neurobiology*.

[2] Taupin P. (2006). Neurogenesis and Alzheimer's disease. *Drug Target Insights*.

[3] Spalding K. L., Bergmann O., Alkass K. (2013). Dynamics of hippocampal neurogenesis in adult humans. *Cell*.

[4] Marutle A., Ohmitsu M., Nilbratt M., Greig N. H., Nordberg A., Sugaya K. (2007). Modulation of human neural stem cell differentiation in Alzheimer (APP23) transgenic mice by phenserine. *Proceedings of the National Academy of Sciences of the United States of America*.

[5] Lazarov O., Marr R. A. (2010). Neurogenesis and Alzheimer's disease: at the crossroads. *Experimental Neurology*.

[6] Mu Y., Gage F. H. (2011). Adult hippocampal neurogenesis and its role in Alzheimer's disease. *Molecular Neurodegeneration*.

[7] Rodríguez J. J., Verkhratsky A. (2011). Neurogenesis in Alzheimer's disease. *Journal of Anatomy*.

[8] Einstein O., Ben-Hur T. (2008). The changing face of neural stem cell therapy in neurologic diseases. *Archives of Neurology*.

[9] Jin K., Xie L., Mao X. O., Greenberg D. A. (2006). Alzheimer's disease drugs promote neurogenesis. *Brain Research*.

[10] Lilja A. M., Luo Y., Yu Q.-S. (2013). Neurotrophic and neuroprotective actions of (−)- and (+)-phenserine, candidate drugs for Alzheimer's disease. *PLoS ONE*.

[11] Lilja A. M., Röjdner J., Mustafiz T. (2013). Age-dependent neuroplasticity mechanisms in Alzheimer Tg2576 mice following modulation of brain amyloid-beta levels. *PLoS ONE*.

[12] Lithner C. U., Hedberg M. M., Nordberg A. (2011). Transgenic mice as a model for Alzheimer's disease. *Current Alzheimer Research*.

[13] Mustafiz T., Portelius E., Gustavsson M. K. (2011). Characterization of the brain *β*-amyloid isoform pattern at different ages of Tg2576 mice. *Neurodegenerative Diseases*.

[14] Stewart S., Cacucci F., Lever C. (2011). Which memory task for my mouse? A systematic review of spatial memory performance in the Tg2576 Alzheimer's mouse model. *Journal of Alzheimer's Disease*.

[15] Unger C., Svedberg M. M., Yu W.-F., Hedberg M. M., Nordberg A. (2006). Effect of subchronic treatment of memantine, galantamine, and nicotine in the brain of Tg2576 (APPswe) transgenic mice. *Journal of Pharmacology and Experimental Therapeutics*.

[16] Lahiri D. K., Chen D., Maloney B. (2007). The experimental Alzheimer's disease drug posiphen [(+)-phenserine] lowers amyloid-beta peptide levels in cell culture and mice. *Journal of Pharmacology and Experimental Therapeutics*.

[17] Maccecchini M. L., Chang M. Y., Pan C., John V., Zetterberg H., Greig N. H. (2012). Posiphen as a candidate drug to lower CSF amyloid precursor protein, amyloid-*β* peptide and *τ* levels: target engagement, tolerabilityand pharmacokinetics in humans. *Journal of Neurology, Neurosurgery and Psychiatry*.

[18] Feuerbach D., Lingenhoehl K., Olpe H.-R. (2009). The selective nicotinic acetylcholine receptor *α*7 agonist JN403 is active in animal models of cognition, sensory gating, epilepsy and pain. *Neuropharmacology*.

[19] Feuerbach D., Nozulak J., Lingenhoehl K., McAllister K., Hoyer D. (2007). JN403, in vitro characterization of a novel nicotinic acetylcholine receptor *α*7 selective agonist. *Neuroscience Letters*.

[20] Brannen C. L., Sugaya K. (2000). In vitro differentiation of multipotent human neural progenitors in serum-free medium. *NeuroReport*.

[21] Garthe A., Huang Z., Kaczmarek L., Filipkowski R. K., Kempermann G. (2014). Not all water mazes are created equal: cyclin D2 knockout mice with constitutively suppressed adult hippocampal neurogenesis do show specific spatial learning deficits. *Genes, Brain and Behavior*.

[22] Blurton-Jones M., Kitazawa M., Martinez-Coria H. (2009). Neural stem cells improve cognition via BDNF in a transgenic model of Alzheimer disease. *Proceedings of the National Academy of Sciences of the United States of America*.

[23] Bruno M. A., Leon W. C., Fragoso G., Mushynski W. E., Almazan G., Cuello A. C. (2009). Amyloid *β*-induced nerve growth factor dysmetabolism in Alzheimer disease. *Journal of Neuropathology & Experimental Neurology*.

[24] Capsoni S., Cattaneo A. (2006). On the molecular basis linking Nerve Growth Factor (NGF) to Alzheimer's disease. *Cellular and molecular neurobiology*.

[25] Laske C., Stransky E., Leyhe T. (2006). Stage-dependent BDNF serum concentrations in Alzheimer's disease. *Journal of Neural Transmission*.

[26] Michalski B., Fahnestock M. (2003). Pro-brain-derived neurotrophic factor is decreased in parietal cortex in Alzheimer's disease. *Molecular Brain Research*.

[27] Peng S., Wuu J., Mufson E. J., Fahnestock M. (2005). Precursor form of brain-derived neurotrophic factor and mature brain-derived neurotrophic factor are decreased in the pre-clinical stages of Alzheimer's disease. *Journal of Neurochemistry*.

[28] Ethell I. M., Ethell D. W. (2007). Matrix metalloproteinases in brain development and remodeling: synaptic functions and targets. *Journal of Neuroscience Research*.

[29] Koshimizu H., Kiyosue K., Hara T. (2009). Multiple functions of precursor BDNF to CNS neurons: negative regulation of neurite growth, spine formation and cell survival. *Molecular Brain*.

[30] Lee R., Kermani P., Teng K. K., Hempstead B. L. (2001). Regulation of cell survival by secreted proneurotrophins. *Science*.

[31] Autry A. E., Monteggia L. M. (2012). Brain-derived neurotrophic factor and neuropsychiatric disorders. *Pharmacological Reviews*.

[32] Lu Y., Christian K., Lu B. (2008). BDNF: a key regulator for protein synthesis-dependent LTP and long-term memory?. *Neurobiology of Learning and Memory*.

[33] Faigle R., Song H. (2013). Signaling mechanisms regulating adult neural stem cells and neurogenesis. *Biochimica et Biophysica Acta—General Subjects*.

[34] Li J.-Y., Englund E., Holton J. L. (2008). Lewy bodies in grafted neurons in subjects with Parkinson's disease suggest host-to-graft disease propagation. *Nature Medicine*.

[35] Kihara T., Shimohama S., Sawada H. (1997). Nicotinic receptor stimulation protects neurons against beta-amyloid toxicity. *Annals of Neurology*.

[36] Lilja A. M., Porras O., Storelli E., Nordberg A., Marutle A. (2011). Functional interactions of fibrillar and oligomeric amyloid-*β* with alpha7 nicotinic receptors in alzheimer's disease. *Journal of Alzheimer's Disease*.

[37] Liu Q., Zhao B. (2004). Nicotine attenuates beta-amyloid peptide-induced neurotoxicity, free radical and calcium accumulation in hippocampal neuronal cultures. *British Journal of Pharmacology*.

[38] Liu Y., Hu J., Wu J. (2012). *α*7 nicotinic acetylcholine receptor-mediated neuroprotection against dopaminergic neuron loss in an MPTP mouse model via inhibition of astrocyte activation. *Journal of Neuroinflammation*.

[39] Verkhratsky A., Olabarria M., Noristani H. N., Yeh C.-Y., Rodriguez J. J. (2010). Astrocytes in Alzheimer's Disease. *Neurotherapeutics*.

[40] Carter S. F., Schöll M., Almkvist O. (2012). Evidence for astrocytosis in prodromal alzheimer disease provided by 11C-deuterium-L-deprenyl: a multitracer PET paradigm combining 11C-Pittsburgh compound B and 18F-FDG. *Journal of Nuclear Medicine*.

[41] Shen J.-X., Yakel J. L. (2012). Functional *α*7 nicotinic ACh receptors on astrocytes in rat hippocampal CA1 slices. *Journal of Molecular Neuroscience*.

[42] Counts S. E., He B., Che S. (2007). *α*7 nicotinic receptor up-regulation in cholinergic basal forebrain neurons in Alzheimer disease. *Archives of Neurology*.

[43] Ikonomovic M. D., Wecker L., Abrahamson E. E. (2009). Cortical *α*7 nicotinic acetylcholine receptor and *β*-amyloid levels in early Alzheimer disease. *Archives of Neurology*.

[44] Bednar I., Paterson D., Marutle A. (2002). Selective nicotinic receptor consequences in APP_SWE_ transgenic mice. *Molecular and Cellular Neuroscience*.

[45] Yu W.-F., Guan Z.-Z., Bogdanovic N., Nordberg A. (2005). High selective expression of alpha7 nicotinic receptors on astrocytes in the brains of patients with sporadic Alzheimer's disease and patients carrying Swedish APP 670/671 mutation: a possible association with neuritic plaques. *Experimental Neurology*.

[46] Liu Q., Xie X., Lukas R. J., St.John P. A., Wu J. (2013). A novel nicotinic mechanism underlies *β*-amyloid-induced neuronal hyperexcitation. *Journal of Neuroscience*.

[47] Nilbratt M., Porras O., Marutle A., Hovatta O., Nordberg A. (2010). Neurotrophic factors promote cholinergic differentiation in human embryonic stem cell-derived neurons. *Journal of Cellular and Molecular Medicine*.

[48] Wicklund L., Leão R. N., Strömberg A.-M. (2010). Beta-amyloid 1-42 oligomers impair function of human embryonic stem cell-derived forebrain cholinergic neurons. *PLoS ONE*.

[49] Court J. A., Lloyd S., Johnson M. (1997). Nicotinic and muscarinic cholinergic receptor binding in the human hippocampal formation during development and aging. *Developmental Brain Research*.

