# Supplementary material for: Neural Stem Cell Transplant-Induced Effect on Neurogenesis and Cognition in Alzheimer Tg2576 Mice Is Inhibited by Concomitant Treatment with Amyloid-Lowering or Cholinergic α7 Nicotinic Receptor Drugs
Source: Neural Plast. 2015 Jul 15;2015:370432. doi: 10.1155/2015/370432 (PMC4518185; doi:10.1155/2015/370432)
Supplement: Supplementary file 1 — JN403 exerts neuroprotective effects on hNSC-derived neurons in culture Neurotrophic actions of the α7 nAChR partial agonist JN403 and the Aβ modulatory drug (+)-phenserine were assessed by examining the survival, differentiation and maturation of progenitor cells in vitro. JN403 protected hNSC-derived βIII-tubulin marked neurons against brain extract from a patient with AD containing various Aβ species (Fig. S1A). On the other hand, JN403 or (+)-phenserine did not significantly alter the number and/or pattern of MAP2-positive Tg2576 cortical neurons in culture (Fig. S1B). The swimming velocity and the total distance the mice swam during the acquisition period did not differ significantly between the groups Hippocampal-dependent spatial learning and memory in Tg2576 mice (n=6) and age-matched non-transgenic littermates (wild type, n=6) were tested in the MWM navigation task. The swimming velocity and the total distance the mice swam during the acquisition period did not differ significantly between transgenic and wildtype animals (Figures S2A and S2B). Tg2576 mice (vehicle- or hNSC-transplanted and saline- or drug-treated), aged 6–9 months, underwent 60-second trials 4 times a day at 15-second intervals for 5 days. Swimming velocity or the total distance swum did not differ among Tg2576 mice in the various groups (Figure S2C and S2D). (+)-Phenserine treatment reduces Aβ40 levels in the brains of hNSC-transplanted Tg2576 mice In this study, a reduction in Aβ40 levels was measured in the frontal cortices and hippocampi of hNSC-transplanted Tg2576 mice treated with (+)-phenserine, but not in the cohort of mice treated with JN403 or saline. In comparison to sham-transplanted, saline-treated mice, a significant reduction in Aβ40 levels, but not in Aβ42 levels, was measured in the frontal cortex (29 % reduction, p<0.05, Dunn's test, Figure S3A) and hippocampus (42 % reduction, p<0.05, Dunn's test, Figure S3B) of hNSC-transplanted mice treated with (+)-phenserine. No signi [file 370432.f1.pdf]

Supplemental information

Figure S1. JN403 exerts neuroprotective actions on hNSC-derived neurons in culture. (A) Expression of neuronal marker βIII-tubulin (red) and glial marker GFAP (green) in hNSCs *in vitro* after 28 days' differentiation. The hNSCs were untreated (CTRL), or exposed weekly to brain extracts from a healthy control (HC), a patient with AD (AD), AD+JN403 (JN), or AD +(+)-phenserine (PHEN) Representative images of CTRL, AD, and AD+JN are outlined in (A), representative of two independent experiments with four replicate samples in each. (B) Expression of the neuronal marker MAP2 (red) and the number of branch points in untreated Tg2576 transgenic mouse primary cortex neurons in culture (CTRL) and after weekly exposure to JN403 (JN) or (+)-phenserine (PHEN) for 21 days. The data are expressed as means ± SEM. Images of CTRL, JN and PHEN are representative of three biological replicates. $^{**}p<0.01$ compared with CTRL, $^{\#}p<0.05$, $^{\#\#}p<0.01$ compared to AD.

Figure S2. Swimming velocity and total distance swum by wildtype and Tg2576 mice in the Morris water maze. (A) Swimming velocity and **(B)** total distance swum by wild type (wt, n=6) and Tg2576 (APPswe, n=6) mice in the Morris water maze (MWM) during 4 days of acquisition training. **(C)** Swimming velocity and **(D)** total distance swum in the MWM during 5 days of acquisition training in the follow-up for sham (vehicle)-transplanted, saline-treated (SHAM+SAL) Tg2576 mice, or mice receiving hNSC transplants and saline (hNSC+SAL), JN403 (hNSC+JN), or (+)-phenserine (hNSC+PHEN).

Figure S3. (+)-Phenserine treatment in combination with hippocampal hNSC transplantation decreases brain Aβ_{40} levels in Tg2576 mice. (A) Cortical and **(B)** hippocampal levels of Aβ_{40} (pmol/g tissue) measured by a sandwich ELISA assay were significantly reduced by (+)-phenserine treatment in hNSC-transplanted Tg2576 mice (hNSC+PHEN) compared to saline-treated mice receiving sham (vehicle) transplantation (SHAM+SAL). JN=JN403. *$p<0.05$ compared to SHAM+SAL. The data are expressed as ± SEM.

Figures

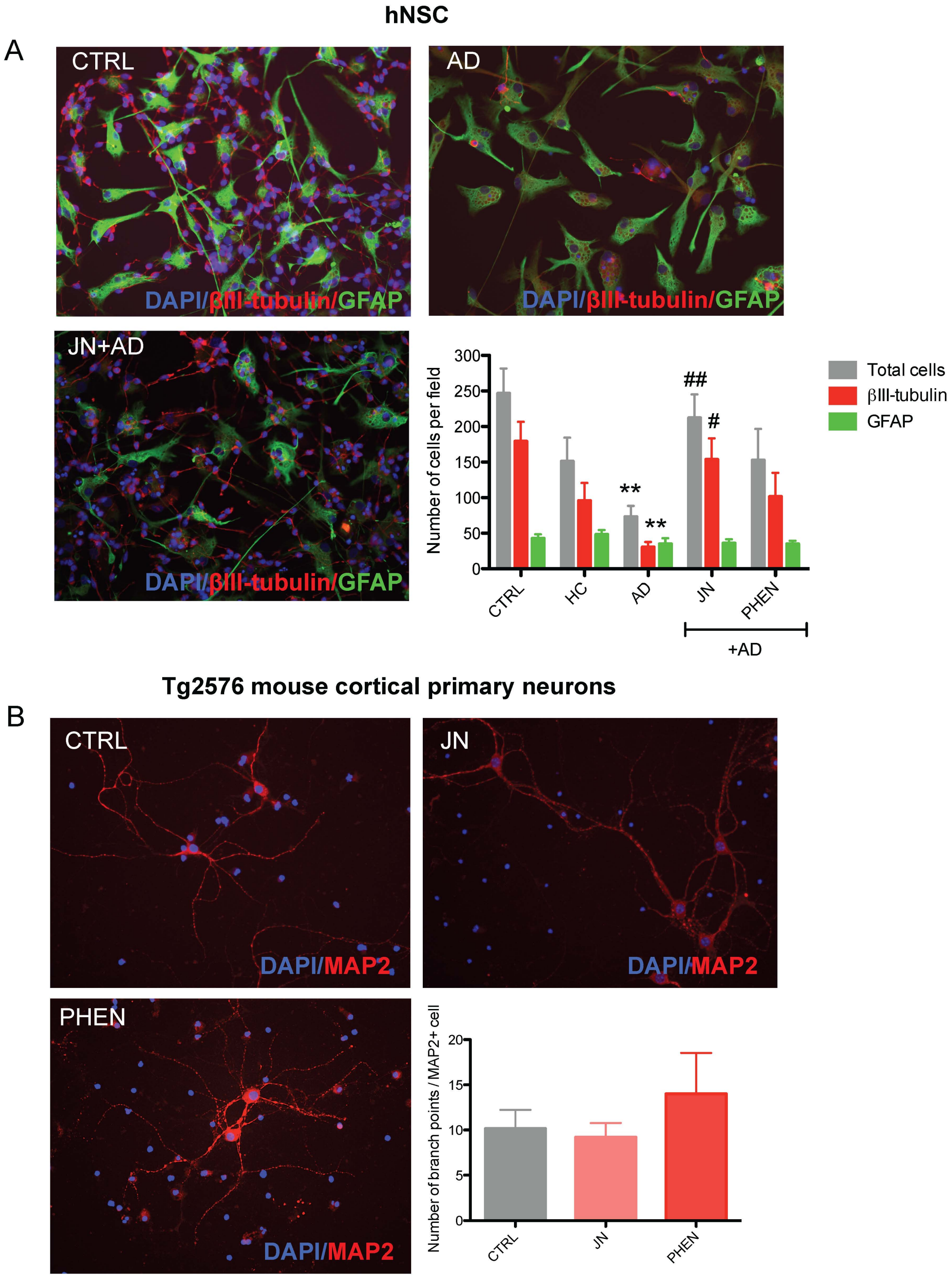

hNSC
A
CTRL
AD
JN+AD
DAPI/βIII-tubulin/GFAP
Number of cells per field
300
250
200
150
100
50
0
##
#
**
**
CTRL
HC
AD
JN
PHEN
+AD
Total cells
βIII-tubulin
GFAP
Tg2576 mouse cortical primary neurons
B
CTRL
JN
PHEN
DAPI/MAP2
Number of branch points / MAP2+ cell
20
15
10
5
0
CTRL
JN
PHEN

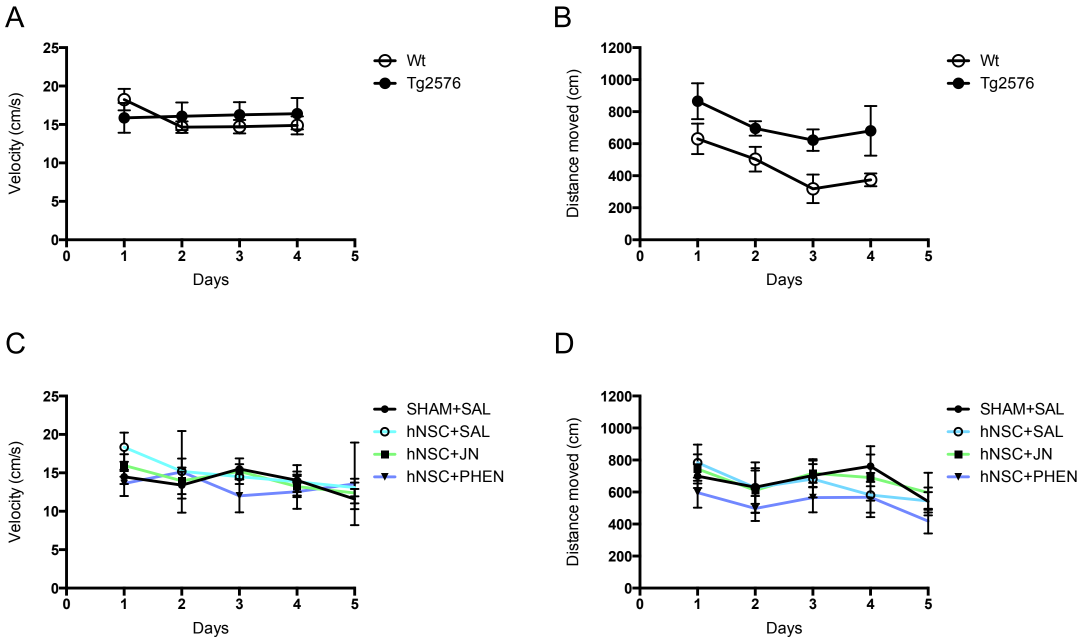
A
B
C
D
Velocity (cm/s)
Distance moved (cm)
Days
Wt
Tg2576
SHAM+SAL
hNSC+SAL
hNSC+JN
hNSC+PHEN

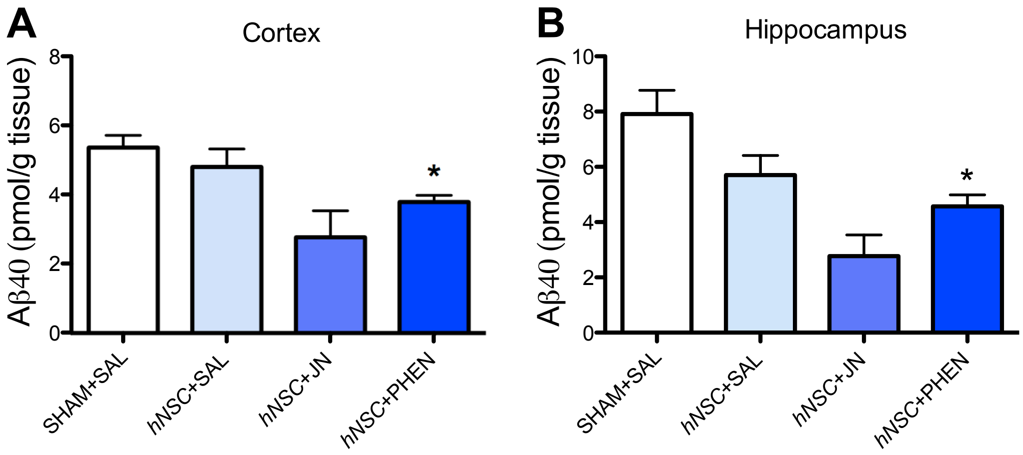
A
Cortex
B
Hippocampus
Aβ40 (pmol/g tissue)
*
SHAM+SAL
hNSC+SAL
hNSC+JN
hNSC+PHEN

Supplementary Table 1. Tg2576 mice included in the transplantation study according to treatment, number of animals per group, age and sex. The numbers of animals that underwent the Morris Water Maze navigation task are included in brackets. * One animal was omitted from the Morris water maze analysis, since it could not be detected by the automated video-tracking system due to its fur color. Abbreviations: human neural stem cell (hNSC), vehicle transplanted + saline treatment (SHAM+SAL), hNSC transplanted + saline treated (hNSC+SAL), hNSC transplanted + (+)-phenserine treated (hNSC+PHEN), and hNSC transplanted + JN403 treated (hNSC+JN).

**Treatment**	**Number of mice**	**Age (median, min-max)**	**Sex (M/F)**
SHAM+SAL	9	7 (6-9)	3/6
hNSC+SAL	9* (8)	7 (6-7)	3/6
hNSC+PHEN	7* (6)	7 (7-9)	2/5
hNSC+JN	5	7 (7-9)	2/3
